# Leaf-size control beyond transcription factors: Compensatory mechanisms

**DOI:** 10.3389/fpls.2022.1024945

**Published:** 2023-01-23

**Authors:** Hiromitsu Tabeta, Shizuka Gunji, Kensuke Kawade, Ali Ferjani

**Affiliations:** ^1^ Department of Life Sciences, Graduate School of Arts and Sciences, The University of Tokyo, Tokyo, Japan; ^2^ Department of Biology, Tokyo Gakugei University, Tokyo, Japan; ^3^ RIKEN Center for Sustainable Resource Science, Yokohama, Japan; ^4^ National Institute for Basic Biology, Okazaki, Japan; ^5^ Department of Basic Biology, School of Life Science, Graduate University for Advanced Studies (SOKENDAI), Okazaki, Japan

**Keywords:** *Arabidopsis thaliana*, leaf morphogenesis, cell proliferation, post-mitotic cell expansion, compensation, cell-autonomous, non-cell-autonomous

## Abstract

Plant leaves display abundant morphological richness yet grow to characteristic sizes and shapes. Beginning with a small number of undifferentiated founder cells, leaves evolve *via* a complex interplay of regulatory factors that ultimately influence cell proliferation and subsequent post-mitotic cell enlargement. During their development, a sequence of key events that shape leaves is both robustly executed spatiotemporally following a genomic molecular network and flexibly tuned by a variety of environmental stimuli. Decades of work on *Arabidopsis thaliana* have revisited the compensatory phenomena that might reflect a general and primary size-regulatory mechanism in leaves. This review focuses on key molecular and cellular events behind the organ-wide scale regulation of compensatory mechanisms. Lastly, emerging novel mechanisms of metabolic and hormonal regulation are discussed, based on recent advances in the field that have provided insights into, among other phenomena, leaf-size regulation.

## 1 Introduction

Pioneering studies on Arabidopsis unveiled the basis of genetic control of major plant organs, such as leaves, flowers, and roots (Irish, 2010; Petricka et al., 2012; González et al., 2012; Kalve et al., 2014). Although most developmental events and concomitant cellular processes have been described thoroughly, it is only during the past few decades that we have integrated the molecular pathways (i.e. relating key genes, receptors, sensors, etc.) behind plant morphogenesis.

Leaf emergence and polarity acquisition and the differentiation of the various cell types and tissues have been described in detail (Asnacios and Hamant, 2012; Lau and Bergmann, 2012; Du et al., 2018; Ali et al., 2020; Vercruysse et al., 2020; Gorelova et al., 2021; Liu et al., 2021). Yet, the core molecular framework behind leaf-size regulation remains unclear. We argue that this relates to a conserved feature of growing organs in seed plants: compensation, a phenomenon whereby cell size compensates for cell division in the establishment of organ size. This is what this review focuses on, taking the leaf as a model system.

## 2 Compensation: One phenotype, several means

Leaves are typically flat. They capture sunlight, and convert carbon dioxide into carbohydrates by photosynthesis to sustain the plant autotrophic lifestyle. Also, plant leaves are polarized, possessing two structurally different sides, the adaxial and abaxial sides, which are specialized for light capture/photosynthesis and gas exchange, respectively (Bowman et al., 2002; Dkhar and Pareek, 2014; Dow and Bergmann, 2014; and the references therein). Hence, plant leaves can be viewed as expandible structural units, which emerge and grow to highly reproducible predetermined sizes and shapes, which are often used as traits in taxonomy (Tsukaya, 2003). Plant leaves, in general, lack a self-renewing meristem and thus cannot grow indefinitely. Leaf development usually proceeds *via* initiation, growth, and maturation stages (Tsukaya, 2002a; Kalve et al., 2014; Tsukaya, 2014; Tsukaya, 2018).

Leaf cells can divide only for a fixed period of time, which is crucial to determine the number of cells within the leaf. Then, their proliferative ability is gradually lost, as they enter a second stage of post-mitotic cell differentiation marked by a considerable increase in cell size accompanied by increased vacuole volume and cell wall synthesis (Johnson and Lenhard, 2011; González et al., 2012; Powell and Lenhard, 2012). Leaf size is reproducible under controlled light, temperature, and nutritional regimes. In addition, size increase in plants is an irreversible process. That is because morphogenesis in the plant kingdom, unlike the animal kingdom, does not rely on cell contractility, cell migration and cell death. Therefore, leaves are also a simpler model system to decipher what makes organs stop growing upon reaching an appropriate size (Beemster et al., 2006; Micol, 2009; Tsukaya, 2018).

Molecular genetics have identified a plethora of genes contributing to leaf-size control (González et al., 2012; Hepworth and Lenhard, 2014). This has led to draw an overall picture of the gene regulatory network operating during leaf development (González and Inzé, 2015; Wang et al., 2021). The dynamic interactions among key genetic components have recently been uncovered. For instance, DELLA proteins repress gibberellin signaling to modulate cell proliferation and expansion (Sun and Gubler, 2004; de Lucas et al., 2008; Achard et al., 2009). Such DELLA-mediated growth suppression is executed by GROWTH REGULATORY FACTOR (GRF) transcriptional factors at least in cold stress response (Lantzouni et al., 2020). GRFs act within a core module to primarily orchestrate cell proliferation together with GRF INTERACTING FACTOR (GIF) transcriptional co-activators including GIF1/ANGUSTIFOLIA3 (AN3) (Kim and Kende, 2004; Horiguchi et al., 2005; Kim and Tsukaya, 2015; Liebsch and Palatnik, 2020). This module is also connected downstream of TCP transcriptional factors and their targeting microRNA miR319 (Rodriguez et al., 2010; Schommer et al., 2014), which are recognized as important components of leaf lamina growth (Nath et al., 2003; Efroni et al., 2013; Das Gupta and Nath, 2015; Challa et al., 2021; Rath et al., 2022). Although the above findings delineate the hierarchical organization and interconnections among the genetic network governing leaf-size control, this topic has already been covered with an increasing pace. A more exhaustive synthesis of our current knowledge can be found elsewhere (Vercruysse et al., 2020).

In the simplest scenario, leaf size would be defined as the linear function of cell number and cell size. However, in leaf primordia, failure in the proliferative stage to produce sufficient cells triggers excessive cell expansion, the so-called compensation (Tsukaya, 2002b; Beemster et al., 2003; Beemster et al., 2006; Tsukaya, 2008). Because cell division precedes cell differentiation (which also involves cell expansion) in a region confined to the leaf primordia basal part, the proliferative stage likely generates intrinsic signals that affect the final cell size during the following differentiation stage. This poses a question of how the above cellular processes, which occur in distinct regions of leaf primordia, are coordinated during development. Given the importance of cell division and expansion in leaf-size control, compensation has emerged as a key phenomenon to uncover how the interconnection between cell division and expansion is achieved. However the molecular basis behind compensation remains unclear.

Large scale genetic screening has uncovered some of the compensatory mechanisms through the identification of a large number of mutants and transgenics displaying compensation (**Table 1**; Horiguchi et al., 2005; Horiguchi et al., 2006a; Horiguchi et al., 2006b; Fujikura et al., 2007a, Fujikura et al., 2007b, Fujikura et al., 2009). For instance, kinematic analyses of cell size dynamism unveiled the presence of three classes of compensation, based on cell expansion mode (Ferjani et al., 2007). More specifically, while class I has an enhanced post-mitotic cell expansion rate (seen in *angustifolia* [*an*]*3-4*, *fugu2-1*/*fasciata* [*fas*]*1-6*, and *erecta*[*er*]*-102*); class II has an extended post-mitotic cell expansion period (seen in *fugu5-1*, *icl-2*, *mls-2*, *pck1-2*, and *ibr10*), and class III has an increased size of dividing cells (seen in *KIP-RELATED PROTEIN 2* [*KRP2*] overexpressing plants) (De Veylder et al., 2001; Ferjani et al., 2007; Ferjani et al., 2008; Ferjani et al., 2010; Ferjani et al., 2013a; Ferjani et al., 2013b; Ferjani et al., 2014a; Ferjani et al., 2014b; Katano et al., 2016; Takahashi et al., 2017; Tabeta et al., 2021).

**Table 1:** List of compensation-exhibiting mutants, transgenic plants and their related suppressors.

ORF number	Gene name	Type of mutation	Reference	Compensation category	Suprossor of CCE
AT1G68310	*AE7*	Loss-of-function	Yuan et al. (2010)		
AT5G28640	*AN3/GIF1*	Loss-of-function	Kim and Kende (2004) Horiguchi et al. (2005)	Class I	*XS2/CCX4* (Fujikura et al., 2020)
AT4G37750	*ANT*	Loss-of-function	Mizukami and Fischer (2000)		
AT3G48750	*CDKA;1*	Dominant negative	Hemerly et al. (1995)		
AT4G34160 AT5G67260 AT3G50070	*CYCD3*	Loss-of-function of *CYCD3;1CYCD3;2CYCD3;3*	Dewitte et al. (2007)		
AT2G26330	*ER*	Loss-of-function	Horiguchi et al. (2006b) Ferjani et al. (2007)	Class I	
AT2G40550	*ETG1*	Loss-of-function	Takahashi et al. (2008)		
AT5G64630	*FAS2*	Loss-of-function	Exner et al. (2006)		
*	*FUGU1*	Recessive mutation	Ferjani et al. (2007)		
AT1G65470	*FUGU2/FAS1*	Loss-of-function	Exner et al. (2006); Ferjani et al. (2007); Ramirez-Parra and Gutierrez (2007); Hisanaga et al. (2013)	Class I	
*	*FUGU3*	Dominant mutation	Ferjani et al. (2007)		
*	*FUGU4*	Dominant mutation	Ferjani et al. (2007)		
AT1G15690	*FUGU5*	Loss-of-function	Ferjani et al. (2007); Ferjani et al. (2011)	Class II	*ECH2*; *IBR1*,*3*,*10; VHA-a2* and *VHA-a3* (Katano et al., 2016; Takahashi et al., 2017; Tabeta et al., 2021; Nakayama et al., 2022)
AT2G26300	*GPA1*	Loss-of-function	Ullah et al. (2001)		
AT4G14430	*IBR10*	Recessive mutation	Tabeta et al. (2021)	Class II	*ECH2* (Katano et al., 2016; Tabeta et al., 2021)
AT3G21720	*ICL*	Loss-of-function	Takahashi et al. (2017)	Class II	*ECH2* (Katano et al., 2016; Takahashi et al., 2017)
AT2G23430	*KRP1/ICK1*	Over-expression	Wang et al. (2000)		
AT3G50630	*KRP2*	Over-expression	De Veylder et al. (2001); Ferjani et al. (2007)	Class III	*DET3* (Ferjani et al., 2013a; Ferjani et al., 2013b)
AT5G48820	*KRP3*	Over-expression	Jun et al. (2013)		
AT2G42620	MAX2	Loss-of-function	Horiguchi et al. (2006b)		
AT5G03860	*MLS*	Loss-of-function	Takahashi et al. (2017)	Class II	*ECH2* (Katano et al., 2016; Takahashi et al., 2017)
AT2G10606 AT5G35407	*miR396*	Over-expression of *miR396A* or *miR396B*	Liu et al. (2009); Rodriguex et al. (2010)		
AT5G55920 AT3G25520 AT5G39740	*OLI*	Loss-of-function of *OLI2* and *OLI5* or *OLI2* and *OLI7*	Fujikura et al. (2009)		
AT4G37870	*PEPCK*	Loss-of-function	Takahashi et al. (2017)	Class II	*ECH2* (Katano et al., 2016; Takahashi et al., 2017)
AT4G00100	*PFL2*	Loss-of-function	Ito et al. (2000)		
AT4G31700	*RPS6A*	Loss-of-function	Horiguchi et al. (2011)		
AT3G53890	*RPS21B*	Loss-of-function	Horiguchi et al. (2011)		
AT5G64140	*RPS28B*	Loss-of-function	Horiguchi et al. (2011)		
AT1G65660	*SMP*	Epimutation	Clay and Nelson (2005)		
AT3G04740	*SWP*	Loss-of-function	Autran et al. (2002)		
AT2G42260	*UVI4/PYM*	Loss-of-function	Hase et al. (2006)		

* Indicates unpublished and/or unidentified gene.

Taking the above into account, compensation-exhibiting mutants have altered coordination between cell division and expansion (Ferjani et al., 2008; Nakayama et al., 2022). Furthermore, cell-autonomous and non-cell-autonomous pathways have been demonstrated to be implicated in compensation (Ferjani et al., 2008; Kawade et al., 2010; Ferjani et al., 2013a; Ferjani et al., 2014a; Nozaki et al., 2020). Finally, the fact that compensation occurs in a wide range of plant species other than Arabidopsis—including tobacco, rice, snapdragon, and ​​North American lake cress—suggests that the developmental mechanisms that trigger compensation are widely conserved at least in seed plants (Hemerly et al., 1995; Barrôco et al., 2006; Delgado-Benarroch et al., 2009; Horiguchi and Tsukaya, 2011; Amano et al., 2015). However, despite their similar cellular phenotypes, compensation refers to a group of heterogeneous processes driven by different mechanisms (Ferjani et al., 2007; Ferjani et al., 2008; Hisanaga et al., 2015).

## 3 Underpinnings of compensatory coordination between cell division and expansion

**Figure 1:**
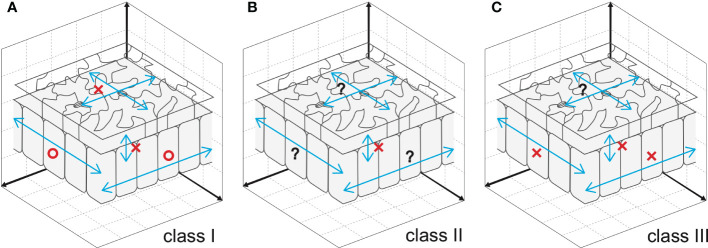
Cellular spatial relationships during induction of CCE. **(A)** Mesophyll cells exploit cell-to-cell communication to stimulate CCE in response to deficient cell proliferation in *an3*, which has class I compensation (Kawade et al., 2010). By contrast, epidermal cells trigger CCE in a cell-autonomous manner, preventing cell-to-cell communication across tissue layers (Nozaki et al., 2020). **(B)** Cell-to-cell communication between epidermal and mesophyll cells is absent in *fugu5*, which has class II compensation (Gunji et al., 2022). Given that excess PPi inhibits metabolic reactions, *fugu5*-mediated induction of CCE is likely controlled in a cell-autonomous manner. **(C)** Direct inhibition of cell cycle progression by KRP overexpression, a representative of class III compensation, induces CCE in a cell- and tissue-autonomous manner. This was determined using chimeric KRP2-overexpressing leaves generated using the Cre/*lox*-P system (Kawade et al., 2010) and KRP1-overexpressing leaves with a tissue-specific expression system (Bemis and Torii, 2007). Epidermal cell-to-cell communication remains to be explored. Circles and crosses indicate, respectively, the presence and absence of cell-to-cell communication that stimulates CCE. Question marks are added when cell-to-cell communication is still untested.

Cell-to-cell communication is an effective way to coordinate cellular processes in time and space and hence to realize stereotyped leaf size. Besides classical anatomy (Szymkowiak and Sussex, 1996), a series of works on the model plant Arabidopsis identified signaling pathways that facilitate cell-to-cell communication (Sieburth et al., 1998; Kim et al., 2003; Serralbo et al., 2006; Savaldi-Goldstein et al., 2007; Eriksson et al., 2010). However, the compensatory interplay between cell proliferation and post-mitotic cell expansion during the induction of compensatory cell enlargement (CCE) is ill-known. Because this knowledge on cellular dynamics is instrumental in determining future directions aiming to delineate the holistic mechanisms of compensation, it has been recently investigated using leaves chimeric for the key elements of class I, II, or III compensation (Kawade et al., 2010; Gunji et al., 2022) (**Figure 1**). The above investigations revealed that both cell-autonomous and non-cell autonomous mechanisms are involved in CCE. In the following sections, we summarize the major outcome of these studies.

## 3.1 *an3*-mediated class I compensation: contribution of plasmodesmata

The Cre/*lox*-P system enables heat shock-dependent induction or suppression of *AN3* expression in an *an3* or wild-type genetic background, respectively (Kawade et al., 2010). When the expression of *AN3* is induced in *an3* subepidermal cells in very-early-stage leaf primordia, an *AN3*-expressing sector forms among the *an3* cell population during leaf development. This leaf chimera exhibited CCE irrespective of cellular genotype (*i.e.*, *an3* and *AN3*-expressing cells). Similar results were obtained when leaf chimeras were generated by patchy deletion of the expression of *AN3* in the wild-type genetic background. These observations indicated that the *an3*-mediated induction of CCE occurs *via* cell-to-cell communication in leaf mesophyll tissue. Although the ability to stimulate CCE propagates in a non-cell-autonomous manner, the signaling range is confined to one half of the leaf partitioned by the midrib (Kawade et al., 2010). In contrast to mesophyll tissue, cell-autonomous behavior to stimulate CCE was observed in epidermal tissue of AN3 leaf chimeras generated using the Cre/*lox*-P system or a tissue-specific expression system (Nozaki et al., 2020). In summary, the cell-to-cell communication that induces CCE in *an3* is cell-type dependent (**Figure 1A**).

It is plausible that this property is attributable to the plasmodesmata aperture size, which varies in time and space to control symplasmic connections (Crawford and Zambryski, 2001; Roberts et al., 2001; Burch-Smith and Zambryski, 2010; Fitzgibbon et al., 2013). Several chaperones and RNA exosomes mediate selective symplasmic transport of signaling molecules, including proteins and mRNAs (Xu et al., 2011; Kitagawa et al., 2022). Apoplastic transport is an alternative pathway for the exchange of signaling molecules between cells. This machinery has been characterized in the context of plant defense, in which bursts of reactive oxygen species (ROS) upon pathogen infection elicit phytohormone signaling (Wendehenne et al., 2014; Noctor et al., 2018). ROS-mediated signaling contributes to the balance between cell proliferation and post-mitotic cell expansion in growing sepals and roots (Tsukagoshi et al., 2010; Tsukagoshi, 2012; Hong et al., 2016; Mabuchi et al., 2018).

In leaf development, the salicylic acid (SA) response is involved in CCE in *an3*. The *extra-small sisters* (*xs*) mutants isolated from a large leaf-size and -shape mutant collection (Horiguchi et al., 2006b) showed normal cell proliferation but compromised post-mitotic cell expansion (Fujikura et al., 2007a). Among them, the *xs2* mutant, which harbors a mutation in a gene encoding the putative endomembrane H^+^-dependent K^+^ transporter CATION CALCIUM EXCHANGER 4 (CCX4), showed ROS overproduction and an elevated SA response (Fujikura et al., 2020). Importantly, eliminating XS2/CCX4 function in the *an3* mutant fully suppressed CCE (Fujikura et al., 2020), indicating crosstalk between CCE and the SA response. This finding provides insight into the mechanism by which cell proliferation and post-mitotic cell expansion are coordinated beyond the cellular level.

Whereas the AN3 protein is capable of moving between cells to promote cell proliferation (Kawade et al., 2013; Kawade et al., 2017), the aforementioned cell-to-cell communication for CCE is observed in the *an3* genetic background. The non-cell-autonomous signaling that activates CCE in *an3*, in addition to the non-cell-autonomous signaling downstream of AN3 protein movement to promote cell proliferation, are both largely unclear. Enhanced knowledge of AN3-related genetic regulatory networks will be instrumental in resolving these issues at the molecular level (Vercruyssen et al., 2014; Nelissen et al., 2015; Zhang et al., 2018; Jun et al., 2019; Fujikura et al., 2020; Hussain et al., 2022).

## 3.2 *fugu5*-mediated class II compensation: contribution of metabolic regulation

Although a factor(s) produced in the mesophyll has been proposed as a signal that coordinates leaf size *via* cell-to-cell communication (class I), compensation is likely mediated by large-scale metabolic reprogramming in class II. Therefore, from the perspective of cell number and size dynamism, a key task is to identify which metabolic changes contribute to class II-mediated compensation.

For instance, class II is observed in the *fugu5* Arabidopsis mutant, which has lost H^+^-PPase activity, the ability to hydrolyze pyrophosphate (PPi), and concomitant vacuolar acidification (Ferjani et al., 2007; Ferjani et al., 2011; Bertoni, 2011; Kriegel et al., 2015; Segami et al., 2018). This mutation leads to excessive accumulation of PPi in the cytosol, partial inhibition of gluconeogenesis, and a reduction in the content of sucrose (Suc) produced from triacylglycerol (TAG), the major seed storage lipid in Arabidopsis (Ferjani et al., 2011). Consequently, Suc deficiency significantly reduced the cell number in *fugu5* cotyledons and triggered CCE (Ferjani et al., 2007; Ferjani et al., 2008; Ferjani et al., 2011; Ferjani et al., 2014a; Ferjani et al., 2018). Also, excess PPi triggered major developmental (reduced pavement cell shape complexity) and patterning (stomatal distribution and functioning) defects (Asaoka et al., 2019; Gunji et al., 2020). The above indicates that the impact of PPi is broad but specific. TAG-to-Suc conversion is a major metabolic process that fuels seedling establishment (Bewley and Black, 1994; Graham, 2008). Whereas most studies described a role of Suc in hypocotyl elongation in the dark, its impact on aboveground organ development (in light) was described only recently (Henninger et al., 2022).

PPi may trigger several specific cellular responses (Chen et al., 1990; Lundin et al., 1991; Stitt, 1998; Maeshima, 2000; Heinonen, 2001; Ko et al., 2007; Wang et al., 2016). For example, although excess PPi-related phenotypes in palisade tissue were suppressed by an external supply of carbon (such as Suc) or the removal of PPi in the *fugu5* background, Suc supply had no effect on epidermal cell developmental defects (Asaoka et al., 2019; Gunji et al., 2020). Together, the above reports are in line with the assumption that PPi indeed exerts different effects on different plant tissues and cell types and at different developmental stages.

Because PPi is a strong inhibitor of anabolic reactions, several PPases are dedicated to its hydrolysis, preventing accumulation of toxic levels (Segami et al., 2018). H^+^-PPase (*FUGU5*) is a master regulator of cytosolic PPi homeostasis (Ferjani et al., 2011; Kriegel et al., 2015; Asaoka et al., 2016; Fukuda et al., 2016; Ferjani et al., 2018; Asaoka et al., 2019). *fugu5* provided insight into the effect of PPi metabolism on leaf development and their potential crosstalk. In other words, phenotypic dissection of ​​*fugu5* suggested a pivotal role for balanced metabolism during the heterotrophic growth stage, indicating that leaf size is controlled by metabolic networks, with a relatively long-lasting effect. The mechanism is discussed in Section 4.

Genetic studies of the TAG-to-Suc pathway identified several key enzymes whose loss-of-function affected seedling establishment with varying levels of penetrance (Hayashi et al., 1998; Hooks et al., 1999; Froman et al., 2000; Eastmond et al., 2000; Germain et al., 2001; Footitt et al., 2002; Rylott et al., 2003; Fulda et al., 2004). To evaluate the link between TAG-to-Suc conversion and CCE, we phenotypically characterized *icl-2*, *mls-2*, *pck1-2* (Takahashi et al., 2017), and *ibr10* mutants (Tabeta et al., 2021; Tabeta et al., 2022), all of which displayed class II CCE (Katano et al., 2016; Takahashi et al., 2017; Tabeta et al., 2021). Hence, producing Suc from TAG during seed germination is crucial for proper cotyledon development. Yet, based on the high mobility of Suc between leaf cells and tissues, as well as its vital role in plant cells, it is technically challenging to pursue the molecular mechanism of class II CCE by simply restricting Suc production and or tracking its dynamic flux.

H^+^-PPases are implicated in plant growth, development, and PPi homeostasis (Ferjani et al., 2011; Segami et al., 2018). Nonetheless, although the contribution of PPi homeostasis to plant growth and development has been investigated, its effect on different tissues and cell types during the plant lifecycle is unclear (Schilling et al., 2017). Does altered PPi homeostasis act cell-autonomously or non-cell-autonomously to modulate critical cell fates *via* specific metabolic processes?​​

The above hypothesis has been formally tested using a spatiotemporal approach by constructing and analyzing transgenic lines in which PPi has been removed from the epidermis, from palisade tissue cells, or during the 4 days following seed imbibition (Gunji et al., 2022). When the yeast PPase IPP1 was expressed in the epidermis or palisade tissue alone, *fugu5* phenotypes were independently restored (Gunji et al., 2022) (**Figure 1B**). Furthermore, the immediate removal of excess PPi after seed imbibition suppressed CCE of palisade cells but failed to totally rescue the epidermal development defects (Gunji et al., 2022). Next, the impacts of spatial and temporal removal of PPi were investigated by capillary electrophoresis time-of-flight mass spectrometry. This analysis revealed that metabolic profiles are differentially affected among transgenic lines, consistent with an axial role in the central metabolism of gluconeogenesis in CCE (Gunji et al., 2022). These findings not only provide a conceptual framework to unveil metabolic fluctuations within leaf tissues with high spatiotemporal resolution, but also suggest that excess PPi exerts its inhibitory effect *in planta* during the early stages of seedling establishment in a tissue- and cell-autonomous manner. In other words, leaf size is a highly complex trait governed by multiple regulatory layers, in which metabolic regulation represents another fundamental side.

## 3.3 *KRP2* overexpression-mediated class III compensation: contribution of cell cycle regulation

*INHIBITOR/INTERACTOR OF CYCLIN-DEPENDENT KINASES/KIP-RELATED PROTEINS* (*KRPs*) encode cyclin-dependent kinase inhibitors, which block cell cycle progression (Wang et al., 1998; Lui et al., 2000; De Veylder et al., 2001). Constitutive overexpression of individual proteins in this family prematurely arrests the mitotic cell cycle and triggers CCE (De Veylder et al., 2001; Verkest et al., 2005; Ferjani et al., 2007; Jun et al., 2013). The activity that stimulates CCE acts in a cell-autonomous manner, because KRP2 overexpressor cells did not stimulate CCE in the adjacent wild-type cells in leaf chimeras for KRP2 overexpression (Kawade et al., 2010). Notably, epidermis-specific expression of *KRP1* induced a similar phenomenon (*i.e.*, CCE was detected in pavement cells without affecting the subepidermal cells of palisade tissue) (Bemis and Torii, 2007) (**Figure 1C**).

The endogenous KRP2 protein is more abundant in post-mitotic cells than in proliferating cells (Ormenese, 2004; Verkest et al., 2005). Strong *KRP2*-overexpressing lines exhibited a more obvious CCE phenotype compared with their weak-expressing counterparts (Verkest et al., 2005; Ferjani et al., 2013a; Ferjani et al., 2013b). Therefore, KRP2 may enhance post-mitotic cell expansion; however, this was refuted by clonal analysis using the Cre/*lox*-P system. In brief, CCE is undetectable when *KRP2* overexpression is induced after exiting the mitotic cell cycle (Kawade et al., 2010). As mitotic cell size in *KRP2* overexpressers is twice that in the wild type (De Veylder et al., 2001; Ferjani et al., 2007; Ferjani et al., 2013a; Ferjani et al., 2013b), the mechanism by which cells sense their default size to set the timing of mitotic entry is likely perturbed. Given that cell-size homeostasis is generally explained by a sizer, timer, or adder model (Roeder et al., 2012; Wood and Nurse, 2015; Pavelescu et al., 2018; D’Ario et al., 2021), it would be of interest to investigate the involvement of the components of these models in CCE. To corroborate this, we need to quantify leaf cellular dynamics, taking into account the contribution of nutritional resource allocation from seeds, which affects seedling growth in particular (Sizani et al., 2019). Future work aims to clarify how mitotic cells integrate information on cell-size homeostasis, and hence cell proliferation, into cell-autonomous stimulation of CCE.

Clonal analyses enabled the dissection of cellular dynamics, *i.e.*, cell-autonomous or non-cell-autonomous, in class I, II, and III compensation. The results suggest that cell-to-cell communication coordinates cell proliferation and post-mitotic cell expansion in class I but is not a prerequisite in classes II and III. How then does deficient cell proliferation trigger post-mitotic cell expansion within a cell or cell population? The molecular mechanism underlying *fugu5*-mediated compensation is in good agreement with the cell-autonomous nature of cellular metabolic disorders.

## 4 Hormonal regulation of class II response phase: contribution of the phytohormone auxin

**Figure 2:**
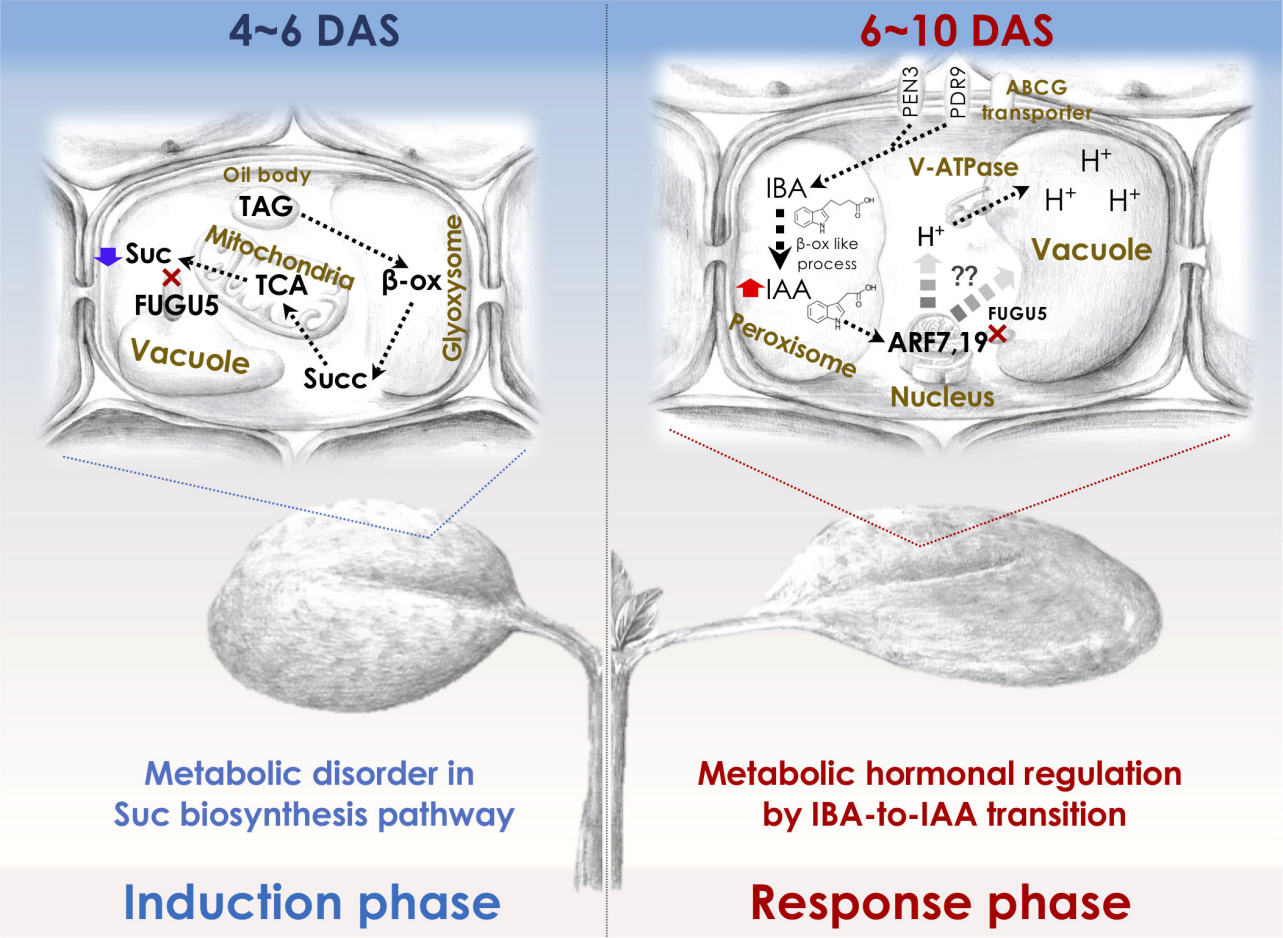
Molecular machinery of the induction and response phases in class II CCE. The decreased cell number (induction phase) in Arabidopsis *fugu5*-mutant cotyledons has been shown to be exclusively due to a decreased level of TAG-derived Suc, and IBA-derived IAA (Katano et al., 2016; Takahashi et al., 2017) has been suggested to mediate CCE (response phase) as follows: First, upon seed imbibition, excess cytosolic PPi in *fugu5* leads to the inhibition of *de novo* Suc synthesis from TAG, a major seed-storage lipid and substrate for the conversion of fatty acids to acetyl-CoA for glyoxylate bypass that takes place in the glyoxysome (Ferjani et al., 2011). This is owing to inhibition of gluconeogenesis (Ferjani et al., 2018). Second, during seedling establishment, metabolic disorder associated with the reduced Suc content (4-6 DAS; left panel) is converted into an ‘output instructive signal’ (6-10 DAS; right panel) that promotes the conversion of IBA, an auxin precursor, to IAA, the natural phytohormone auxin, leading to an increase in endogenous IAA concentration, which is crucial for CCE (Tabeta et al., 2021). Third, increased endogenous IAA (IAA concentration peaks at 8-10 DAS) triggers the TIR/AFB-dependent auxin signaling pathway through the AUXIN RESPONSE FACTORs ARF7 and ARF19, transcriptional activators of early auxin-response genes. This subsequently activates the vacuolar type V-ATPase, leading to an increase in turgor pressure, which ultimately drives an increase in cell size and CCE (Tabeta et al., 2021; Nakayama et al., 2022). β-ox, β-oxidation; Succ, succinate. Glyoxysome, the single membrane-bound organelles that house most of the biochemical machinery required to convert fatty acids derived from TAG to 4-carbon compounds. TCA, the tricarboxylic acid cycle. PEN3, PENETRATION (PEN) 3 is a membrane-localized ATP-binding cassette (ABC) transporter. PDR9, is a member of the pleiotropic drug resistance (PDR) family of ATP-binding cassette transporters. ABCG transporter, G-type ATP-binding cassette (ABCG) transporter. DAS, days after seed sowing.

To elucidate the molecular mechanisms of compensation, it is important to understand the molecular framework of the cell-autonomous induction and response phases for which the *fugu5* mutant (class II compensation) has been used as a prototype. Although the reduced cell number in the induction phase in *fugu5* was attributed to reduced Suc synthesis, CCE in *fugu5* is also controlled metabolically and hormonally (**Figure 2**). In this section, we describe how forward and reverse genetics-based approaches have provided insight into class II CCE (Katano et al., 2016; Tabeta et al., 2021; Nakayama et al., 2022).

By mutagenizing *fugu5* dry seeds using ^12^C^6+^ heavy-ion irradiation, Katano and colleagues performed a large-scale screening using forward genetics to identify key genes in CCE. This phenotypic screening identified ENOYL-CoA HYDRATASE 2 (ECH2) activity as a prerequisite for CCE to occur in the *fugu5* background. Because ECH2 has dual functions (β-oxidation of TAG-to-Suc conversion (Graham, 2008; Li et al., 2019) and conversion of indole-3-butyric acid (IBA) to the auxin 3-indole acetic acid (IAA) (Strader et al., 2011)), these metabolic processes have been postulated to play a major role in CCE. IAA levels are tightly regulated by *de novo* biosynthesis, transportation, and inactive conversion, and IBA has been implicated in cotyledon and root development (Strader et al., 2010; Frick and Strader, 2018). Hence, the suppression of CCE in *fugu5 ech2* was a result of defective IAA biosynthesis from IBA caused by loss of ECH2 activity.

Reverse genetics in the *fugu5* background showed that CCE in the *ibr1 ibr3 ibr10 fugu5* quadruple mutant, which is defective in IBA-to-IAA conversion (Strader et al., 2010; Strader et al., 2011), was totally suppressed (Tabeta et al., 2021). In contrast, *pen3 fugu5* and *pdr9 fugu5*, in which IBA efflux is compromised, exhibited a high-IAA phenotype (Strader and Bartel, 2009; Ruzicka et al., 2010; Aryal et al., 2019) and enhanced CCE (Tabeta et al., 2021). Consistently, endogenous IAA levels were twofold higher in *fugu5* (in 8-10-day-old seedlings). These findings indicate that IAA converted from IBA is essential for CCE, in agreement with the finding that the degree of CCE reflects the intracellular IAA level (Tabeta et al., 2021). How is high auxin sensed, interpreted, and transduced into CCE in *fugu5*?

AUXIN RESPONSE FACTORS (ARF) 7 and 19 and V-ATPase activity are essential for CCE in *fugu5*. More specifically, *arf7-1 arf19-1 fugu5-1* and *vha-a2 vha-a3 fugu5-1* triple mutants did not exhibit CCE, despite having a significantly reduced number of cells (Tabeta et al., 2021). The vacuole accounts for the majority of the cell volume and is essential for cell enlargement, in class II CCE, proton translocation *via* V-ATPase contributes to vacuole enlargement and promotes cell-size control *via* the ARF7 ARF19 module. The above findings are valid for all other mutants with class II CCE, namely *isocitrate lyase* (*icl*; Eastmond et al., 2000), *malate synthase* (*mls*; Cornah et al., 2004), *phosphoenolpyruvate carboxykinase1* (*pck1*; Penfield et al., 2004), and *ibr10* (Zolman et al., 2008), but not *an3* and *fas1* (class I compensation) (Katano et al., 2016; Takahashi et al., 2017; Tabeta et al., 2021). Therefore, IBA-related hormonal signaling is likely activated in response to metabolic disorders of the central metabolism. If so, one major question arises: Could this rather indicate that auxin integrates more signals (metabolism, cell cycle, vacuole volume, cell wall remodeling etc.) and thus is at the same hierarchical level as compensation (i.e., integrating heterogeneous pathways)?

Finally, although auxin signaling plays a major role in class II, the phytohormone SA has been proposed to relate to class I CCE in the *an3-4* mutant (Fujikura et al., 2020). These findings indicate that the response phase in classes I and II is under phytohormonal control. Importantly, hormonal cross-talk controlling leaf development has also been discussed. As mentioned above, gibberellins together with DELLA proteins, which are downstream of the IAA response, modulate cell proliferation and expansion (Sun and Gubler, 2004; de Lucas et al., 2008; Achard et al., 2009). Also, some TCPs have been reported to regulate auxin homeostasis and cytokinin by altering the expression of auxin biosynthetic enzymes (Lucero et al., 2015). Since IBA-derived IAA provides NO signaling and promotes other hormonal responses (Fattorini et al., 2017), hormonal cross-talk regulation might also have a role in leaf size regulation in compensation-exhibiting mutant background. To this end, compensation-mediated leaf size control may represent a good starting point for further studies aiming to unveil the broader picture of phytohormonal regulation.

## 5 Environmentally-induced compensation

Growth and development of plants are greatly affected by environmental changes. Because plants cannot move, altering their own tissue structure and concomitant organ size and/or shape to adapt to the ambient environment is critical for their survival. For instance, heterophylly, which is defined as a leaf-form alteration triggered by the surrounding environment, is commonly observed in aquatic and amphibious plants (Li et al., 2019). *Rorippa aquatica* is an amphibious plant found in riparian environments, such as the bank of a natural watercourse including lakes, ponds, and streams, in North America. *R. aquatica* shows a remarkable heterophylly, and develops deeply dissected narrow leaves under submerged conditions whereas it develops simple shaped leaves on terrestrial conditions. The leaf shape changes also in response to varying ambient temperature, in which lower temperature induces dissected narrow leaves. Recently, it was shown that both submergence (Sakamoto et al., 2022) and low-temperature (Amano et al., 2015) treatments caused an increase in cell size in the sub-epidermal palisade tissue layer in mature leaves, along with a decrease in leaf blade area. This phenotype in which cell size increase occurs in the background of tissue-size reduction resembles compensation. Indeed, the expression of some of the compensation-related genes is altered under low-temperature or submergence in *R. aquatica*.

Because phytohormones usually reflect the environmental status, in some ways they represent the secondary messengers of environmental cues. Therefore, it is not surprising to see compensation being dependent on the environment. In other words, in the case of *R. aquatica*, environmental cues may have triggered compensation *via* a yet unidentified hormonal regulatory pathway. These observations also indicate that compensation could be induced not only in mutants, transgenics, and γ-ray–treated plants, but also as an adaptive response to a wide range of environmental stimuli. Together, these findings provide evidence that compensation is a universal phenomenon that is also seen in nature, whereby hormones are acting as downstream instructive signal of the environmental status.

## 6 Outstanding questions and future prospects

Large-scale genetic screening identified a number of genes whose loss- or gain-of-function alters final leaf size. For decades, this prompted work on the morphogenesis of Arabidopsis. Subsequent functional analyses of the above genetic factors revealed the molecular events governing leaf development. Furthermore, bioinformatics techniques identified several key transcription factors (TFs) relevant to core genetic modules (Ichihashi et al., 2014; Sinha et al., 2016). However, our understanding of leaf-size control is incomplete.

Most developmental events are interpreted based on TFs (Kaufmann and Airoldi, 2018; and references therein). Although TFs are crucial in orchestrating organogenesis, other regulatory factors with more indirect effects have been overlooked. This is a result of the inherent bias of most genetic screening approaches, which identify major non-redundant players, overlooking more indirect and diffuse properties that build on more complex interactions (Nakayama et al., 2022). This includes understudied players (e.g., metabolic molecules) and understudied systemic interactions (e.g., feedback loops). Thus, the compensatory mechanism of leaf-size control cannot be explained by transcriptional regulation processes only (Ferjani et al., 2008; Ferjani et al., 2014a; Ferjani et al., 2018). Multi-omics enables identification of novel small molecules (including metabolic components and regulators) critical in major developmental transitions during plant growth (Omidbakhshfard et al., 2021) and leaf development (Tabeta et al., 2021). Systems biology may also help to identify, in the topology of the molecular network, key events and interactions, to explain how compensation emerges.

Beyond molecule identity and interactions, the mechanical properties of plant cells and tissues should be regarded as an integral part of developmental signaling pathways. Advances in quantitative plant biology have allowed experimentation and modeling using plants (Autran et al., 2021). Hence, the regulatory mechanisms at the crossroads of metabolism, morphogenesis, and mechanics should be integrated with genetic mechanisms to understand the multimodal drivers of leaf-size control (Trinh et al., 2021; Nakayama et al., 2022).

Finally, if compensation is a general and primary size-regulatory mechanism in plant leaves, does it involve, by default, a proprioceptive sensing step? Does CCE generate an additional instructive mechanical signal? If so, how is such a signal perceived, resolved, integrated, and executed to guarantee proper size? To address these questions, leaf development should be reexamined from the perspectives of biomechanics and metabolism.

## Author contributions

HT, SG, KK and AF drafted and wrote the paper. All authors contributed to the article and approved the submitted version.
